# The History of Ancient and Modern Wines

**Published:** 1826-04

**Authors:** 


THE
### MEDICO-CHIRURGICAL
### REVIEW.

VOL. VIII.] *Analytical Series.* [No. 24.

" Nec tibi quid liceat sed quid fecisse decebit
" Occurrat, mentemque domat respectus honesti."

VOL. IV.] APRIL 1, 1826. [No. 8.

[NEW SERIES.]

### I.

*The History of Ancient and Modern Wines.* By ALEXANDER HENDERSON, M.D. Quarto, pp. 408. London, 1824.

Vina parant animos faciuntque caloribus aptos:
Cura fugit, multo diluiturque mero.

WINE is the whip, stimulus, or goad, which causes lagging life to jog on more *speedily*—perhaps more *merrily*. A physical agent of such power is well deserving of the physician's attention. It has attracted the notice of poets and philosophers in all ages. Galen wrote largely on wine, but that portion of his works has not come down to posterity. Homer, Horace, and other poets, ancient and modern, have made the welkin ring with its praises. It makes a great deal of work for the doctors, who are ungrateful enough to be always speaking ill of their best friend. It is assuredly a wonderful compound of contrary qualities:—a stimulant and a sedative—an incentive to virtue and vice—it makes the coward brave—and renders the brave merciful or mad, according to the whim or impulse of the moment. It tends to elicit *truth*, as is evinced by the old adage, *in vino veritas*—while, at the very same moment, it prompts to the enunciation of the most extravagant fictions—hence it is the very soul of poetry. It produces a host of diseases, and is an active agent in the cure of them, like the celebrated weapon whose rust healed the wound inflicted by its point. In short, a large volume would not be sufficient to contain a history of the moral and physical agency of wine.

In the present piping times of peace and plenty, when our invalids spread themselves over the Continent for change of air —and for the pleasure of roaming, after dinner, over the "*carte des vins*"—and when the vineyards of France, Germany, and Italy, pour their tides of nectar through every hotel and alehouse of Old England, it is as incumbent on the medical practitioner to study *vinology* as pathology—if not more so! The nature of his diet, and the kind of wine which he is to drink, if not the first, are certain to be the last questions which the invalid asks, before he pays his fee to the physician, who ought to be ready with his answer. It is true that the disciples of Abernethy make short work of it, like the Lord Chancellor, by issuing an *injunction*,* which is sometimes read, but seldom obeyed. The antediluvian beverage is not to the taste of many patients, who generally prefer Sauterne to sarsaparilla—and Burgundy to the "black draught." It is not much to be wondered at indeed, if, in viewing the former, they should wish for a neck as long as that of a crane, for the purpose of prolonging the pleasure of drinking—while, in eyeing the "doctor's stuff," they should curse Mr. Weiss for not contriving an artificial *swallow*, among his other ingenious *inventions*.

The splendid and costly volume before us is not adapted, nor, we suppose, intended for the profession. There was a time, indeed, when the apothecary's shop was the place to get *wine* in this country; but those good old times of substantial pleasures are gone for ever—and the wines of ipecacuan, antimony, and meadow saffron, are all that remain in the cellars of the pharmacopolist!

Dr. Henderson has drunk deep—if not of ancient wines—at least of ancient lore. With Cato, Varro, and Columella, he has "insensibly entered into the spirit of their agricultural pursuits —accompanied them, as it were, into the fields—visited their plantations—inspected their flocks—watched the progress of their crops—and partaken of the pleasing anxieties of their harvests—returning with them to their dwellings, and becoming familiar with the details of their domestic economy." As for us, unlucky wights, we have little time for such pleasing retrospective rambles. Sufficient for the day is the evil thereof! We dare not quaff, even in imagination, the "rich, potent, and unadulterate" MARONIAN, with which Ulysses fuddled the ferocious Polyphemus in the cave—nor the rosy PRAMNIAN which Hecamede mulled with spices for our professional ancestor, MACHAON, when wounded in the shoulder, and with which the venerable Nestor did not disdain to moisten his clay.—

* "See page 72 of my work."

——————The Nymph of form divine,
Pours a large portion of the Pramnian Wine;
This for the wounded Prince the Dame prepares—
*The cordial beverage reverend Nestor shares.*

It is not for us weak-headed mortals to touch the *fiery* Falernian,* of which Horace wished to broach an Amphora as old as himself—nor can we hope to dilute our inky Port with "water from the choicest springs, conveyed from a great distance by means of majestic aqueducts." Alas, no! We sneer at the delicacy of the Hindoo, who slakes his thirst at the same tank where his neighbour is sacrificing to Cloacina; but what shall we say to the delicate citizens of London, who fill *their tanks* and stomachs with water from the Thames, at that very spot into which one hundred thousand cloacæ, containing every species of filth, and all unutterable things, are daily disgorging their hideous and abominable contents!†

From such revolting ideas, we would fain turn, with our learned author, to feast our imaginations on the banquets of the ancients—not of the Homeric warriors, each of whom was his own *butcher*, (which was natural enough) and sometimes his own shoemaker—but of the lordly Romans, reclined on couches of gold and silver, "inlaid with ivory, tortoiseshell, or the precious metals," quaffing their wines (cooled with snow from the summit of Mons Albanus) out of "goblets richly bedecked with gems."

"Nam virro, ut multi, geminos ad pocula transfert
"A digitis." *Juvenal*, *v.* 43.

* ——————quis puer ocius
Restinguet *ardentis* Falerni
Pocula prætereunte lympha?—*Horace.*

† It is absolutely astonishing that, in these days of refinement, and in a metropolis whose inhabitants pride themselves on delicacy and cleanliness, a practice should obtain, at which posterity will shudder, if they can credit it! We do not believe that a parallel instance of bestial dirtiness can be cited from any part of the globe! A time must come when the people of London will open their eyes to this scene of corruption, veiled and concealed as it is by iron tubes and stony pavements. We are not among the idolators of the ancients; but we do admire the delicacy of their taste in expending so much labour and wealth in commanding abundant supplies of pure and salubrious water for the everlasting city. The New River and the Hampstead waters are, it is true, æthereal streams, as compared with those of Chelsea; but even *these* are little better than a *dilute solution* of dead dogs, cats, and a thousand other animal and vegetable substances in a state of putrefaction!

It is difficult to say how far health may be affected by drinking from such a polluted source, but surely such deleterious substances, however minutely divided, cannot be salubrious. It is, therefore, possible, nay probable, that part of the insalubrity of the city, as compared with the country, may be owing to this cause.

But we dare not venture to transgress farther on the time and patience of our *sober* and serious readers. We trust that we will not be accused of indulging often in themes that are not directly applicable to the every-day business of our lives; yet a moment's digression, now and then, may be excused by way of *relief* to the monotonous tract of "art and science."

Modern Wines. Different countries, and different parts of the same country, produce differences in their wines, according to the kind of vine, the nature of the soil, the climate, and the cultivation of the grape. Dr. Henderson adopts the division of wines into two principal classes, viz. red and white, which he again separates into two orders, dry and sweet, the *genera* depending on distinctive characters derived from soil and climate;—the *species* on particular localities;—and the *varieties* on the respective qualities of the different growths. In addition to the above scheme, there must be introduced a kind of intermediate order between the sweet and dry wines—namely, such wines as have a full body and rich flavour, inclining more to a saccharine, than an austere taste, yet not exactly resembling either. This order embraces a great majority of the fine wines of France, and to them the term *mellow* may be applied.

The soil of France exhibits every species of strata that is most congenial to the vine—the whole expanse of that country, as Chaptal triumphantly observed, from the banks of the Rhine to the foot of the Pyrennees, presenting a succession of fertile and beautiful vineyards, where the most agreeable wines in Europe are grown. The growths of Champagne, Burgundy, Dauphiny, and Bourdeaux, are now decidedly the best which France supplies—the wines of the more northern departments being of an inferior order, and scarcely sufficing, in quantity, for the consumption of the inhabitants.

1. *Champagne.* The growths of this province are divided into river and mountain, or white and red. Champagne is a sparkling or frothing liquor—subjected to an imperfect fermentation, and containing a quantity of carbonic acid gas, generated during the insensible fermentation in bottle, and disengaged on drawing the cork. The district in question, however, furnishes many excellent wines, red and white, which do not effervesce. Most of the white, or Marne River wines, indeed, are brisk, and are superior to the red or Mountain in flavour and aroma, in agreeable pungency, slight acidulous taste—and last, not least, their exhilarating virtues. But the briskest wines are not always the best. They are defective in true vinous quality, and the small portion of alcohol which they contain, immediately escapes from the froth as it rises on the surface, carrying with it the aroma, and leaving the liquor in the glass nearly vapid. "Hence the still, or the creaming, or the slightly sparkling Champagne wines, are more highly valued by connoisseurs, and fetch greater prices, than the full frothing wines." By icing these wines before they are used, the tendency to effervesce is, in some degree, repressed.

The first in rank, among the white wines of Champagne, is the far famed SILLERY, a title derived from a marquess of that name. It is a dry, still liquor, of a light amber colour, with considerable body, and a flavour somewhat analogous to that of the first growths of the Rhine—and being one of the best fermented Champagne wines, may be drunk with safety. It is usual to drink it iced.

Of the Reims Mountain (or red) wines, the CLOS ST. THIERRY "furnishes the only red wine that can be said to unite the rich colour and aroma of Burgundy, with the delicate lightness of Champagne." All these wines, white and red, when well made and placed in cool cellars, will retain their good qualities from ten to twenty years.

2. *Burgundy.* Towards the beginning of the last century, a violent controversy was raised among the faculty of Champagne and Burgundy, respecting the superiority of the wines of these two provinces, and was kept up at intervals till the year 1778, when a verdict was given in favour of Champagne. "It is, however, certain," says Dr. Henderson, (and we entirely agree with him) "that the growths of Burgundy must, in some respects, be considered as the more perfect of the two. In richness of flavour and perfume, and all the more delicate qualities of the juice of the grape, they unquestionably rank as the first in the world; and it was not without reason that the Dukes of Burgundy, in former times, were designated "LES DUCS BONS VINS." They are produced in greatest variety, abundance, and excellence, in the departments of the Côte d'Or, Yonne, Saone, and Loire. The principal vineyards are situated between Dijon and Chagny. The choice *red* growths are the Promanée, Conti, Clos-Vougeôt, Chambertin, Richebough, &c. The *white* vines of Burgundy are less numerous and less known in this country, but maintain the highest rank among the French white wines. At Poligny is grown the famous MONT RACHET wine, surpassing all others of the CÔTE D'OR in high perfume and agreeable nutty flavour.*

* We well remember the delight we experienced in quaffing, at Poligny,

3. *Hermitage—Côte Rôtie.* On the granite hill, that rises five or six hundred feet behind the town of Tain, on the left bank of the Rhone, twelve miles from Valence, are the famous vineyards of the Hermitage. The slope faces the South, and enjoys the full benefit of the sun's rays during the greater part of the day. The *red* Hermitage wines are distinguished by their full body, dark purple colour, exquisite flavour and perfume, which is not unaptly compared to that of raspberry. They ought not to be bottled till they are eight or ten years in the cask. In bottle they keep long, and it is only then that their more delicate qualities are fully developed.

In the lower portion of the Lyonais, the wines of Côte Rôtie take the lead. In flavour and perfume they resemble the Hermitage more than any other growth.

4. We must pass over the delicious wines raised in Languedoc, Provence, and other situations bordering on the shores of the Mediterranean, in which figure the Muscadine and Frontignan wines. The latter is known from all others by the very marked flavour of the grape. The aroma has been compared to that of the elder flower.

5. *The Bordelais, or Claret Wines.* About thirteen leagues to the north of Bourdeaux, the celebrated Medor wine district commences, and extends along the banks of the Gironde and Garonne to within a short distance of the last-mentioned city. Here we have La Fitte, Latour, Barsac, Château Margôt, &c. which figure on every carte de vin through France.

The Bourdeaux wines were celebrated by Ausonius, and none have maintained more uniform repute than the Clarets of the Bordelais. At present they rank as the most perfect which France produces. They keep extremely well, and are improved by sea voyages, hence they are favourite wines throughout all the hotter regions of the earth, wherever Commerce waves her flag. They are much less disposed to acidity and other disorders than the wines of Burgundy, though they contain much less alcohol. The *red* Bordelais wines are in greatest demand for the English market. "There is even a particular manufacture, called *travail à l'Anglais,* which consists in adding to

---

a bottle of this wine, one beautiful evening in August, while preparing to mount the first stupendous pass into the Jura Mountains. This pass was cut by Buonaparte to facilitate the transmission of troops into Switzerland, and on to the Semplon. It was through this same pass that the Austrian army descended from the Jura down upon the fertile plains of France in 1812!

each hogshead of Bourdeaux wine three gallons of Alicant or Benicarlo, half a gallon of Stein wine, and sometimes a small quantity of Hermitage. This mixture undergoes a slight degree of fermentation; and when the whole is sufficiently fretted in, it is exported under the name of Claret."

We have thus taken a rapid survey of the leading characters of the French wines. The genera, species, and varieties, would require a large volume for delineation.

*Spanish Wines.* Spain, like Italy, produces the grape as well as the olive. The range of mountains that overlook her extensive coasts and bound her principal rivers, present the happiest exposures, and the greatest variety of soil for the cultivation of the vine. In the preparation of dry white wines and certain species of sweet wines, the Spaniards stand unrivalled; but the red wines are often spoiled in the fermentation, and are generally dull and heavy on the palate. We cannot, however, afford space to notice more than the famous Xeres or Sherry, the produce of Andalusia, near Cadiz, and so much prized in this country. The vineyards are now principally in the hands of English and Frenchmen, which has led not only to improvement in the wines, but to an abolition of some abominable dirty practices in the manufacture of them. Sherry wine, as every body knows, is now considered the most wholesome of all wines.

*Portugal Wines.* Lisbon and Oporto wines are those most used in England. The banks of the river Douro, for 50 miles from Oporto, furnish the great supply of John Bull's darling Port—which, by the way, is getting much out of repute among the rising generation of wine-bibbers—or rather, as their forefathers would have termed them, milk and water drinkers. The practice of *colouring* these wines with various materials, and *strengthening* them with brandy, is duly descanted on by Dr. Henderson, but it need not detain us here.

*German Wines.* Germany and Hungary present an endless variety of excellent wines. The romantic banks of the Rhine and its contributory streams, whose hills are "crown'd with castle, moat, and tower," afford, in great abundance, that delicious beverage the Hock, so universally admired in this country, especially in the summer. The traveller, in ascending or descending the Rhine, beholds extensive ranges of vineyards, almost all the way from Bonn to Bingen, yielding a profusion of excellent wines, supporting a numerous population, and giving an air of richness and animation to the scenery, which forms an agreeable contrast to the ruins of feudal magnificence that crown the principal heights. The vintages of the Rhinegaw, extending along the right bank of the river, from Mentz to Rudesheim, are the most celebrated. A notion prevails that the Rhenish wines are naturally acid; and the inferior sorts doubtless are so—but this is not the general character of the Rhenish wines which, in good years, have not any perceptible acidity. Their chief distinction is their extreme durability, in which they are not surpassed by any other species of wine. This conservative power evidently does not reside in the spirituous principle of these liquors, for they contain little more than half as much alcohol as is usually found in Madeira imported into this country. This quantity too, is often reduced by long keeping, so low as seven or eight per cent. The saccharine matter is fully decomposed, for it is not found in these wines. Our author thinks that it is to the free tartaric acid that they owe their power of long keeping.

Of the Rhenish wines, the sovereign is that of Johannisberg, which formerly only entered the stomachs of the Monks, and now is confined to that of Prince Metternich, to whom the vineyard belongs. Next in rank is the Steinberg. Then comes the Rudesheimer wine, which grows on the hill opposite to Bingen. The wines of Grafenberg, Markebrunne, &c. will delight the palates of those who journey along the Rhine—as well as those who journey over the bottle at their own fire-sides.

Of the Hungarian wines, which are very numerous, the "Imperial TOKAY" is the most noted, and is prepared from picked and half-dried grapes.

Of the wines of Madeira, the Canaries, and the Cape, we know enough in this country.

Our author touches on the important subject of the adulteration of wines, quoting the humorous passage from Addison's Tatler, respecting this practice in his days. The subterranean philosophers employed in the transmutation of liquors, have no occasion *now* to "squeeze Bourdeaux out of the sloe, and draw Champagne from an apple," whereby they verified the remarkable prophecy of Virgil—

> "The ripening grape shall hang on every thorn"—

for the reduction of duty on Cape wines has enabled these adepts to employ, as occasion may require, a more substantial and convenient menstruum for their preparations than was formerly used. By mixing Cape wines with the lees of other kinds, and tinting and compounding them with various drugs, they manage to counterfeit the more costly vintages of Spain,

Portugal, and even France. They cannot, indeed, deceive the connoisseur in wines, for the vile odour of Cape wine adheres to it under every possible form. The laws of this, and of many other countries, have laid strong prohibitions on the adulteration of wine with many things that are harmless or even salutary, while they pass unnoticed the introduction of very deleterious substances into wines! Dr. Henderson thinks that the use of *litharge*, to correct the acidity of some light wines, as those of the Rhine, Moselle, or the Cape, is not altogether unknown in this country. The presence of that mineral may be easily detected, by adding to such wines, a solution of the hydrosulphuret of potash, or water impregnated with sulphuretted hydrogen gas, which gives a black precipitate. The Spaniards and Dutch are said to fine their wines with oxymuriate of mercury, and even arsenic. It is astonishing how custom reconciles the palate to qualities in wine, which, in themselves, are very repulsive. Thus we are pleased with the coarse acerbity and astringency which Port wine too often derives from the addition of alum, sloes, or oak-bark. We disdain the combination of odoriferous herbs with which the ancients scented their inferior wines, while our own are not unfrequently perfumed by an admixture of nitrous ether!

The last chapter of our author's work is on the dietetic and medicinal qualities of wine. And here Dr. Henderson indulges in some flowery morality, not, however, unsuitable to the subject. He remarks that the gifts of Bacchus are very unequally distributed among his votaries—that the unalloyed (Dr. H. strangely enough calls them *unallayed*) joys are unsubstantial and fleeting in their nature—while the sorrows are real and permanent, and generally become the portion of his most ardent idolators. "They revel, for a time, in feverish gaiety, but the period at length arrives when the dream of happiness dissolves, and they awake to melancholy and despair. Doomed, for the remainder of their days, to endure the anguish of remorse and irremediable disease, they discover, when it is too late, that, in the pursuit of false pleasure, they have drained the cup of life to its bitterest dregs." The picture of the effects of intemperance on the mind is equally cheerless. "Perception is blunted—imagination decays—the memory and judgment are enfeebled—the temper becomes irritable and gloomy —and a degree of moral callousness is superinduced, which steels the heart against all the tender feelings and refined sympathies of our nature!" This is an affecting portraiture, but, fortunately, it does not even generally apply, except to the most beastly drunkard, and particularly to the drinker of ardent spirits. Even in worse times than the present, the effects of wine-drinking were seldom such as are represented above—and, in the present day, there is little danger of such excesses as can ruin the health. It is, indeed, gratifying to observe the general, we might say the universal, spirit of temperance which pervades all the upper classes of society, and we augur the very best effects from such spirit on the rising generation.

It is almost superfluous to remark that, as the stimulant power of wine generally corresponds to the quantity of alcohol which it contains, so this stimulant power must be greatly increased in those wines which contain a large proportion of adventitious and imperfectly combined spirit. The ancients seldom ventured to drink their wine undiluted with water. "What then," says our author, "would they have said, had they witnessed the taste of modern times, and of this nation in particular, which, not content with the most potent vintages that can be extracted from the grape, seeks to give them a fictitious strength by the further addition of alcohol? That wines thus compounded are rendered doubly injurious to the constitution, is certain."

"But it is not to the brandy alone that the noxious effects of certain wines are to be ascribed. If the original fermentation has been imperfect, or if they contain an excess of acids, particularly the gallic or malic acids, their use becomes highly prejudicial, especially to persons of infirm stomachs. When such wines are placed within the temperature of the human body, a renewal of the suppressed fermentation will take place; and what little alcohol they have will rather assist than counteract the acidifying process. Hence the unwholesomeness of most of our domestic wines, which are, in general, but imperfectly fermented, and contain a large portion of malic acid and free saccharine matter, and to many of which brandy is added, with the view of increasing their strength. Perhaps, too, the predominant acids may undergo some transmutation in the stomach, which renders their presence still more detrimental." 352.

The following are the few facts which appear, to our author, to be established, with respect to the comparative virtues of some of the principal wines.

"1. Among the brisk wines, those of Champagne, though not the strongest, may be considered as the best; and they are certainly the least noxious, even when drank in considerable quantity. They intoxicate very speedily, probably in consequence of the carbonic acid gas in which they abound, and the volatile state in which their alcohol is held; and the excitement is of more lively and agreeable character, and shorter duration, than that which is caused by any other species of wine, and the subsequent exhaustion less. Hence, the moderate use of such wines has been found occasionally to assist the cure of hypochondriacal affections and other nervous diseases, where the application of an active diffusible stimulus was indicated. They also possess marked diuretic powers. The opinion which prevails, that they are apt to occasion gout, seems to be contradicted by the unfrequency of that disorder in the province where they are made; but they are generally admitted to be prejudicial to those habits in which that disorder is already formed, especially if it has originated from addiction to stronger liquors. With respect to this class of wines, however, it is to be observed that they are too often drunk in a raw state, when, of course, they must prove least wholesome; and that, in consequence of the want of proper cellars, and other causes which accelerate their consumption, they are very rarely kept long enough to attain their perfect maturity. It is also worthy of notice, that, in order to preserve their sweetness and promote effervescence, the manufacturers of Champagne commonly add to each bottle a portion of syrup, composed of sugar-candy and cream of tartar—the highly frothing kinds receiving the largest quantity. Therefore, contrary to the prevailing opinion, when the wine sparkleth in the glass, and "moveth itself aright," it is most to be avoided, unless the attributes of age should countervail all its noxious properties.

"2. The red wines of Burgundy are distinguished by greater spirituosity and a powerful aroma. Owing, perhaps, to the predominance of the latter principle, they are much more heating than many other wines which contain a larger proportion of alcohol. Though, in the time of Louis XIV. they were prescribed in affections of the chest, no physician of the present day would dream of giving them in such cases. The exhilaration, however, which they cause, is more innocent than that resulting from the use of heavier wines. The better sorts may be sometimes administered with advantage in disorders where stimulant and sub-astringent tonics are required. The same observation will apply to the wines of the Rhone, and the lighter red wines of Spain and Portugal.

"3. Possessing less aroma and spirit, but more astringency than the produce of the Burgundy vineyards, the growths of the Bordelais are, perhaps, of all kinds, the safest for daily use; as they rank among the most perfect light wines, and do not excite intoxication so readily as most others. They have, indeed, been condemned by some writers as productive of gout; but I apprehend without much reason. That, with those persons who are in the habit of soaking large quantities of Port and Madeira, an occasional debauch in Claret may bring on a gouty paroxysm, is very possible:—but the effect is to be ascribed chiefly to the transition from a brandied wine to a lighter beverage—a transition almost always followed by a greater or less derangement of the digestive organs. Besides, we must recollect that the liquor which passes under the denomination of Claret, is generally a compounded wine. It is, therefore, unfair to impute to the wines of the Bordelais those mischiefs which, if they do arise in the manner alleged, are probably, in most instances, occasioned by the admixture of other vintages, of less wholesome quality.

"4. The wines of Oporto, which abound in the astringent principle, and derive additional potency from the brandy added to them previously to exportation, may be serviceable in disorders of the alimentary canal, where gentle tonics are required. But the gallic acid renders them unfit for weak stomachs; and what astringent virtues they shew will be found in greater perfection in the wines of Alicant and Rota, which contain more tannin and less acid. The excitement they induce is of a more sluggish nature than that attending the use of the purer French wines, and does not enliven the fancy in the same degree. As a frequent beverage, they are unquestionably much more pernicious.

"5. For a long time the vintages of Spain, and particularly the *Sacks*, properly so called, were preferred to all others, for medicinal purposes. The wines of Xeres still recommend themselves by the almost total absence of acidity.

"6. Of all the strong wines, those of Madeira, when of good quality, seem the best adapted to invalids; being equally spirituous as Sherry, but possessing a more delicate flavour and aroma, and, though often slightly acidulous, agreeing better with dyspeptic habits. Some have thought them beneficial in cases of atonic gout, probably without much cause; for, whenever a disposition to inflammatory disorders exists, the utility of any sort of fermented liquors is very doubtful.

"7. The lighter wines of the Rhine, and those of the Moselle, are much more refrigerant than any of the preceding, and are frequently prescribed in the countries where they grow, with a view to their diuretic properties. In certain species of fever, accompanied by a low pulse and great nervous exhaustion, they have been found to possess considerable efficacy, and may certainly be given with more safety than most other kinds; as the proportion of alcohol in them is small, and its effects are moderated by the presence of free acids. They are also said to be of service in diminishing obesity.

"8. It is difficult to conjecture on what circumstances the ancients founded their belief in the innocuous qualities of sweet wines, contrasted with the drier and more fully fermented kinds. They may not intoxicate so speedily, and, as they cloy sooner on the palate, are, perhaps, generally drunk in greater moderation. When new, they are exceedingly apt to disorder the stomach; and, when used too freely, they produce all the same effects as the heavier dry wines. In their more perfect state, they may answer the purpose of agreeable and useful cordials; but, as the excess of saccharine matter retards their stimulant operation, they ought always to be taken in small quantities at a time." 356.

In an Appendix, Dr. Henderson has given a table of the relative proportions of alcohol in different wines, partly from Brande, partly from Prout, and partly from Ziz. We shall glance at only the principal wines, and omit the fractional parts. Champagne contains 12 and a half per cent. of alcohol—Burgundy 14—red Hermitage 12—Côte Rôtie 12—Claret 12—Sauterne 14—Sherry nearly 24—Malaga 18—Port 22—Lisbon

18—Bucellas 18. The Rhenish Wines contain from 8 to 12 or 13 per cent. of alcohol, and from 6 to 12 of acid, equal in strength to the concrete tartaric acid—Tokay, 9 per cent. of alcohol—Etna or Syracuse Wines 30—Marsala 18—Lissa 15—Madeira 22—Malmsy Madeira 16—Tenerieffe 19—Constantia 14.

We must now take leave of our author, after taking this rapid survey of his work, and selecting such information from it as we conceived would be most useful to our readers. It is not a work, indeed, of very great interest to the medical reader, though such as should not be entirely neglected by him. Dr. Henderson has expended a great deal of time and labour, in his researches respecting the wines of the ancients, which might, we humbly conceive, have been better employed or bestowed on other subjects. But every man has the right of disposing of his time as is most agreeable to him; and doubtless our author has, in this instance, followed the bent of his inclination.

